# Stimulation of Erythrocyte Cell Membrane Scrambling by Mushroom Tyrosinase

**DOI:** 10.3390/toxins6031096

**Published:** 2014-03-18

**Authors:** Leonie Frauenfeld, Kousi Alzoubi, Majed Abed, Florian Lang

**Affiliations:** Department of Physiology, Eberhard-Karls-University of Tuebingen, Gmelinstr. 5, 72076 Tübingen, Germany; E-Mails: leonie.frauenfeld9@gmail.com (L.F.); kossai.z@gmail.com (K.A.); dr.magd81@hotmail.com (M.A.)

**Keywords:** phosphatidylserine, tyrosinase, calcium, ceramide, cell volume, eryptosis

## Abstract

**Background**: Mushroom tyrosinase, a copper containing enzyme, modifies growth and survival of tumor cells. Mushroom tyrosinase may foster apoptosis, an effect in part due to interference with mitochondrial function. Erythrocytes lack mitochondria but are able to undergo apoptosis-like suicidal cell death or eryptosis, which is characterized by cell shrinkage and cell membrane scrambling leading to phosphatidylserine-exposure at the erythrocyte surface. Signaling involved in the triggering of eryptosis include increase of cytosolic Ca^2+^-activity ([Ca^2+^]_i_) and activation of sphingomyelinase with subsequent formation of ceramide. The present study explored, whether tyrosinase stimulates eryptosis. **Methods**: Cell volume has been estimated from forward scatter, phosphatidylserine-exposure from annexin V binding, [Ca^2+^]_i_ from Fluo3-fluorescence, and ceramide abundance from binding of fluorescent antibodies in flow cytometry. **Results**: A 24 h exposure to mushroom tyrosinase (7 U/mL) was followed by a significant increase of [Ca^2+^]_i_, a significant increase of ceramide abundance, and a significant increase of annexin-V-binding. The annexin-V-binding following tyrosinase treatment was significantly blunted but not abrogated in the nominal absence of extracellular Ca^2+^. Tyrosinase did not significantly modify forward scatter. **Conclusions**: Tyrosinase triggers cell membrane scrambling, an effect, at least partially, due to entry of extracellular Ca^2+^ and ceramide formation.

## 1. Introduction

Mushroom tyrosinase has been suggested for the use in malignancy [1]. When applied with appropriate substrates, it may generate cytostatic products effective *in vivo* [2,3,4]. Tyrosinase may at least in part be effective by interference with mitochondrial function [2]. On the other hand, mushroom tyrosinase generates products leading to mutagenesis and carcinogenesis [5,6,7,8,9,10]. As a matter of fact, tyrosinase has been shown to trigger the suicidal death of nucleated cells or apoptosis [11].

Even though lacking mitochondria and nuclei, erythrocytes are still able to undergo apoptosis-like suicidal death or eryptosis [12]. Eryptosis may be elicited by increase of cytosolic Ca^2+^ concentration ([Ca^2+^]_i_) resulting at least partially from Ca^2+^ entry through Ca^2+^-permeable cation channels [12]. An increase of [Ca^2+^]_i_ may shrink erythrocytes due to activation of Ca^2+^-sensitive K^+^ channels leading to K^+^ exit, hyperpolarization, Cl^-^ exit and, thus, cellular loss of KCl and osmotically obliged water [13]. Increased [Ca^2+^]_i_ further stimulates cell membrane scrambling with translocation of phosphatidylserine to the erythrocyte surface [12]. The Ca^2+^ sensitivity of cell membrane scrambling is increased by ceramide [12]. Signaling of eryptosis further includes caspases [14,15,16,17,18] and several kinases including AMP activated kinase AMPK [19], casein kinase 1α [20,21], cGMP-dependent protein kinase [22], Janus-activated kinase JAK3 [23], protein kinase C [24], p38 kinase [25], PAK2 kinase [26], as well as sorafenib [27] and sunifinib [28] sensitive kinases.

Eryptosis is stimulated by a wide variety of xenobiotics [12,28,29,30,31,32,33,34,35,36,37,38,39,40,41,42,43,44,45,46,47,48,49,50,51,52,53,54,55,56,57,58,59,60,61,62,63] and excessive eryptosis is observed in several clinical disorders [12], such as diabetes [18,64,65], renal insufficiency [66], hemolytic uremic syndrome [67], sepsis [68], malaria [69], sickle cell disease [70], Wilson’s disease [71], iron deficiency [72], malignancy [73], phosphate depletion [74], and metabolic syndrome [48].

The present study explored, whether tyrosinase influences [Ca^2+^]_i_, cell volume and phosphatidylserine translocation to the erythrocyte surface. The observations disclose that exposure to tyrosinase stimulates erythrocyte cell membrane scrambling, an effect paralleled by and at least in part secondary to increase of [Ca^2+^]_i_.

## 2. Results and Discussion

**Figure 1:**
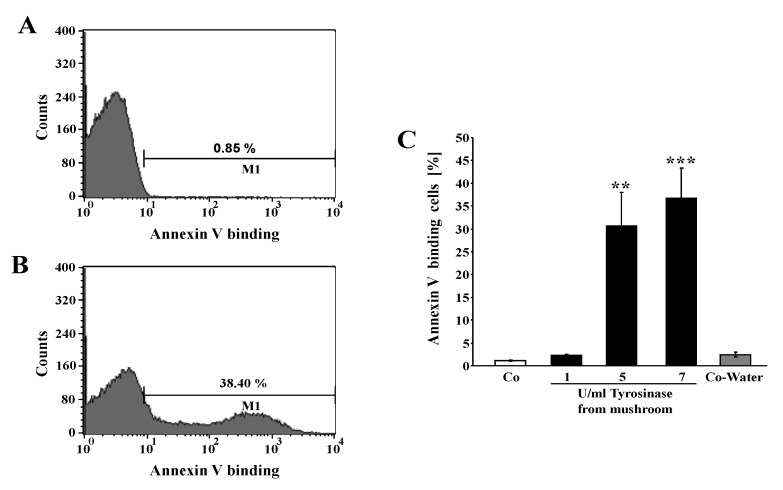
Effect of tyrosinase on phosphatidylserine exposure*.* (**A**,**B**) Original histogram of annexin V binding of erythrocytes following exposure for 24 h to Ringer solution without (**A**) or with (**B**) 7 U/mL tyrosinase. M1 indicates the gating of annexin V binding cells (**C**) Arithmetic means ± SEM (*n* = 4) of erythrocyte annexin-V-binding following incubation for 24 h to Ringer solution without (white bar) or with (black bars) presence of tyrosinase (1–7 U/mL). ** (*p* < 0.01), *** (*p* < 0.001) indicate significant differences from the absence of tyrosinase (ANOVA).

The present study addressed the effect of tyrosinase on eryptosis. A hallmark of eryptosis is the breakdown of phosphatidylserine asymmetry of the erythrocyte cell membrane, which increases the phosphatidylserine abundance at the cell surface. Phosphatidylserine exposing erythrocytes were identified by annexin-V-binding in FACS analysis. As illustrated in Figure 1, a 24-h exposure to tyrosinase increased the percentage of annexin-V-binding erythrocytes, an effect reaching statistical significance at 5 U/mL tyrosinase activity.

**Figure 2:**
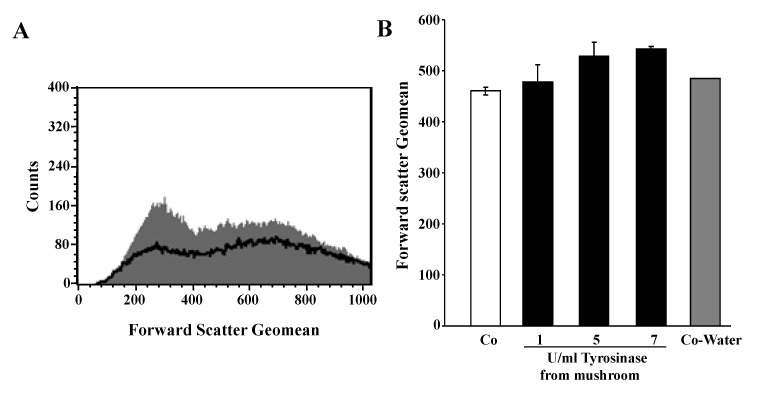
Effect of tyrosinase on erythrocyte forward scatter. (**A**) Original histogram of forward scatter of erythrocytes following exposure for 24 h to Ringer solution without (grey shadow) and with (black line) presence of 7 U/mL tyrosinase; (**B**) arithmetic means ± SEM (*n* = 4) of the normalized erythrocyte forward scatter (FSC) following incubation for 24 h to Ringer solution without (white bar) or with (black bars) tyrosinase (1–7 U/mL).

A second hallmark of eryptosis is cell shrinkage. Accordingly, cell volume was estimated utilizing forward scatter, which was determined by flow cytometry. As shown in Figure 2, a 24-h exposure to tyrosinase tended to increase erythrocyte forward scatter, an effect, however, not reaching statistical significance. Further experiments were performed to elucidate whether tyrosinase abrogates the effect of the Ca^2+^ ionophore inomomycin (1 µM) on erythrocytes forward scatter. As a result, a 30-min exposure of erythrocytes was followed by a decrease of forward scatter from 518 ± 6 (*n* = 4) to 134 ± 5 (*n* = 4) in the absence of tyrosinase and from 557 ± 8 (*n* = 4) to 194 ± 16 (*n* = 4) in the presence of 7 U/mL tyrosinase. Thus, tyrosinase did not abrogate the shrinking effect of excessive Ca^2+^ entry.

**Figure 3:**
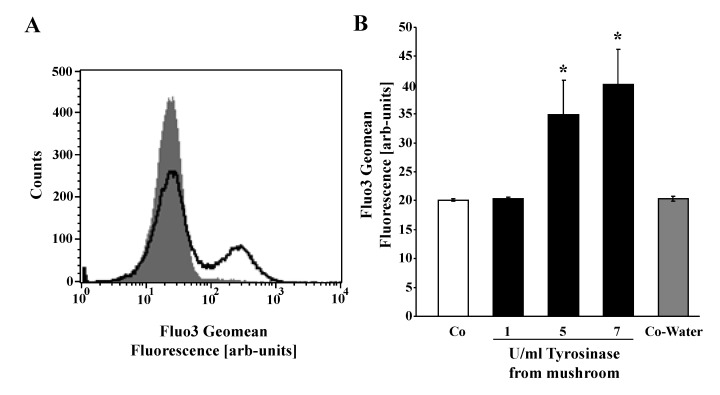
Effect of mushroom tyrosinase on erythrocyte cytosolic Ca^2+^ concentration. (**A**) Original histogram of Fluo3 fluorescence in erythrocytes following exposure for 24 h to Ringer solution without (grey shadow) and with (black line) presence of 7 U/mL tyrosinase; (**B**) arithmetic means ± SEM (*n* = 4) of the Fluo3 fluorescence (arbitrary units) in erythrocytes exposed for 24 h to Ringer solution without (white bar) or with (black bars) tyrosinase (1–7 U/mL). * (*p* < 0.05) indicates significant difference from the absence of tyrosinase (ANOVA).

Cell membrane scrambling is stimulated by increase of cytosolic Ca^2+^ activity ([Ca^2+^]_i_). Thus, [Ca^2+^]_i_ was determined utilizing Fluo3 fluorescence. To this end, erythrocytes were loaded with Fluo3-AM and Fluo3 fluorescence determined in FACS analysis following prior incubation in Ringer solution without or with tyrosinase. As shown in Figure 3, a 24-h exposure of human erythrocytes to tyrosinase was followed by an increase of Fluo3 fluorescence, an effect reaching statistical significance at 5 U/mL of tyrosinase concentration.

**Figure 4:**
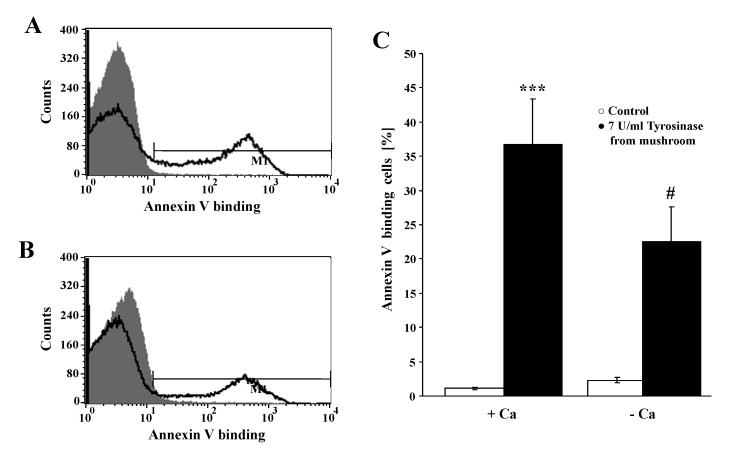
Effect of Ca^2+^ withdrawal on tyrosinase- induced annexin-V-binding. (**A**,**B**) Original histograms of annexin V binding erythrocytes following exposure for 24 h to Ringer solution without (grey shadow) and with (black line) presence 7 U/mL tyrosinase in the presence (**A**) and absence (**B**) of calcium. M1 indicates the gating of annexin V binding cells; (**C**) arithmetic means ± SEM (*n* = 4) of the percentage of annexin-V-binding erythrocytes after a 24 h treatment with Ringer solution without (white bar) or with (black bars) 7 U/mL tyrosinase in the presence (left bars, +Ca) and absence (right bars, −Ca) of calcium. *** (*p* < 0.001) indicates significant difference from the absence of tyrosinase (ANOVA) # (*p* < 0.05) indicates significant difference from the respective values in the presence of Ca^2+^.

An additional series of experiments was performed testing whether extracellular Ca^2+ ^entry was required for the effect of tyrosinase on cell membrane scrambling. Erythrocytes were exposed to 7 U/mL tyrosinase for 24 h, either in the presence of 1 mM Ca^2+^, or in the absence of Ca^2+^, and the presence of Ca^2+^ chelator EGTA (1 mM). As illustrated in Figure 4, the effect of tyrosinase on annexin-V-binding was significantly decreased in the nominal absence of Ca^2+^. However, even in the absence of extracellular Ca^2+^ tyrosinase still increased the percentage annexin V binding erythrocytes.

**Figure 5:**
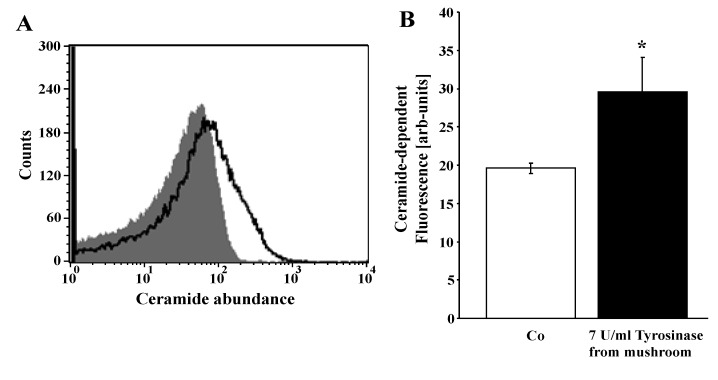
Effect of tyrosinase on ceramide formation. (**A**) Original histogram of ceramide surface abundance of erythrocytes following exposure for 24 h to Ringer solution without (grey shadow) and with (black line) presence of 7 U/mL tyrosinase; (**B**) arithmetic means ± SEM (*n* = 4) of ceramide abundance after a 24-h incubation in Ringer solution without (white bar) or with 7 U/mL tyrosinase (black bar). * (*p* < 0.05) indicates significant difference from the absence of tyrosinase (*t* test).

In order to test whether tyrosinase increased the formation of ceramide, which is known to trigger eryptosis even without increase of [Ca^2+^]_i_, ceramide abundance at the erythrocyte surface was determined utilizing an anti-ceramide antibody. As illustrated in Figure 5, exposure of erythrocytes to 7 U/mL tyrosinase significantly increased the abundance of ceramide at the erythrocyte surface.

The present study uncovers that tyrosinase stimulates cell membrane scrambling leading to phosphatidylserine translocation to the erythrocyte surface. Treatment of human erythrocytes with 5 U/L tyrosinase is further followed by increase of cytosolic Ca^2+^ activity ([Ca^2+^]_i_) and ceramide formation.

Despite its effect on [Ca^2+^]_i_, tyrosinase did not decrease but tended to increase the erythrocyte forward scatter. The increase of [Ca^2+^]_i_ were expected to activate Ca^2+^ sensitive K^+^ channels [12] with subsequent K^+^ exit, cell membrane hyperpolarization, Cl^−^ exit and, thus, cellular loss of KCl with osmotically obliged water [13]. Possibly, tyrosinase inhibits the Ca^2+^ sensitive K^+^ channels, blocks the Cl^−^ channels or stimulates some other mechanism increasing cell volume. Notably, the ionomycin induced erythrocyte shrinkage was not abrogated in the presence of tyrosinase. Excessive erythrocyte swelling may eventually result in rupture of the cell membrane leading to release of cellular hemoglobin, which is filtered in renal glomerula and subsequently occludes renal tubules [75].

Eryptosis is followed by removal of the defective erythrocytes, Phosphatidylserine at the surface of eryptotic cells binds to the respective receptors of phagocytosing cells leading to subsequent engulfment of the affected erythrocytes [12]. Accordingly, eryptotic cells are rapidly cleared from circulating blood [12]. If the accelerated loss of erythrocytes during stimulated eryptosis is not compensated by enhanced formation of new erythrocytes, the clearance of eryptotic erythrocytes from circulating blood results in anemia [12].

Phosphatidylserine exposing erythrocytes may further adhere to the vascular wall by binding of phosphatidylserine at the erythrocyte surface to endothelial CXCL16/SR-PSO [76]. The adherence of suicidal erythrocytes to the vascular wall is expected to interfere with microcirculation [76,77,78,79,80,81]. Phosphatidylserine exposing erythrocytes have further been shown to trigger blood clotting and, thus, foster thrombosis [77,82,83].

## 3. Experimental Section

### 3.1. Erythrocytes, Solutions and Chemicals

Leukocyte-depleted erythrocytes were kindly provided by the blood bank of the University of Tübingen. The study is approved by the ethics committee of the University of Tübingen (184/2003V). Erythrocytes were incubated *in vitro* at a hematocrit of 0.4% in Ringer solution containing (in mM) 125 NaCl, 5 KCl, 1 MgSO_4_, 32 N-2-hydroxyethylpiperazine-N-2-ethanesulfonic acid (HEPES), 5 glucose, 1 CaCl_2_; pH 7.4 at 37 °C for 48 h. Where indicated, erythrocytes were exposed to mushroom tyrosinase (Sigma, Aldrich, Germany) at the indicated concentrations. In Ca^2+^-free Ringer solution, 1 mM CaCl_2_ was substituted by 1 mM glycol-bis(2-aminoethylether)-*N*,*N*,*N*',*N*'-tetraacetic acid (EGTA).

### 3.2. FACS Analysis of Annexin-V-Binding and Forward Scatter

After incubation under the respective experimental condition, 50 µL cell suspension was washed in Ringer solution containing 5 mM CaCl_2_ and then stained with Annexin-V-FITC (1:200 dilution; ImmunoTools, Friesoythe, Germany) in this solution at 37 °C for 20 min under protection from light. In the following, the forward scatter (FSC) of the cells was determined, and annexin-V fluorescence intensity was measured with an excitation wavelength of 488 nm and an emission wavelength of 530 nm on a FACS Calibur (BD, Heidelberg, Germany). Following treatment with Ca^2+^-free Ringer solution, care was taken to measure annexin V binding rapidly enough to avoid triggering of cell membrane by the addition of 5 mM Ca^2+^.

### 3.3. Measurement of Intracellular Ca^2+^

After incubation erythrocytes were washed in Ringer solution and then loaded with Fluo-3/AM (Biotium, Hayward, USA) in Ringer solution containing 5 mM CaCl_2_ and 5 µM Fluo-3/AM. The cells were incubated at 37 °C for 30 min and washed twice in Ringer solution containing 5 mM CaCl_2_. The Fluo-3/AM-loaded erythrocytes were resuspended in 200 µL Ringer. Then, Ca^2+^-dependent fluorescence intensity was measured with an excitation wavelength of 488 nm and an emission wavelength of 530 nm on a FACS Calibur.

### 3.4. Determination of Ceramide Formation

For the determination of ceramide, a monoclonal antibody-based assay was used. After incubation, cells were stained for 1 h at 37 °C with 1 µg/mL anti ceramide antibody (clone MID 15B4, Alexis, Grünberg, Germany) in PBS containing 0.1% bovine serum albumin (BSA) at a dilution of 1:5. The samples were washed twice with PBS-BSA. Subsequently, the cells were stained for 30 min with polyclonal fluorescein isothiocyanate (FITC) conjugated goat anti-mouse IgG and IgM specific antibody (Pharmingen, Hamburg, Germany) diluted 1:50 in PBS-BSA. Unbound secondary antibody was removed by repeated washing with PBS-BSA. The samples were then analyzed by flow cytometric analysis with an excitation wavelength of 488 nm and an emission wavelength of 530 nm.

### 3.5. Statistics

Data are expressed as arithmetic means ± SEM. As indicated in the figure legends, statistical analysis was made using ANOVA with Tukey’s test as post-test and *t-*test as appropriate. n denotes the number of different erythrocyte specimens studied. As different erythrocyte specimens used in distinct experiments are differently susceptible to triggers of eryptosis, the same erythrocyte specimens have been used for control and experimental conditions.

## 4. Conclusions

Tyrosinase stimulates Ca^2+^ entry, which in turn triggers cell membrane scrambling with phosphatidylserine translocation to the erythrocyte surface.
